# Assessing the Predictive Power of Frailty and Life-Space Mobility on Patient-Reported Outcomes of Disability in Older Adults with Low Back Pain

**DOI:** 10.3390/healthcare11071012

**Published:** 2023-04-02

**Authors:** Benyapa Thonprasertvat, Inthira Roopsawang, Suparb Aree-Ue

**Affiliations:** Ramathibodi School of Nursing, Faculty of Medicine Ramathibodi Hospital, Mahidol University, Bangkok 10400, Thailand

**Keywords:** patient-reported outcomes of disability, older adults, low back pain, frailty, life-space mobility

## Abstract

Background: Frailty and decreased life-space mobility are known as risk factors to develop physical limitations leading to disability in older adults with low back pain (LBP). This cross sectional study aimed to investigate the prevalence and predictive power of frailty and life-space mobility on patient-reported outcomes of disability in older adults with LBP. Methods: The sample comprised 165 older adults with LBP who visited two tertiary care hospitals between December 2021 and February 2022. The participants responded to structured standard questionnaires. Data were analyzed using descriptive statistics and robust logistic regression. Results: More than two-thirds of participants were classified as non-frail (26.67%) or pre-frail (66.67%). Mobility restrictions and minimal to severe disability were identified. Controlling other variables, frailty (OR = 1.74, 95% CI: 1.14–2.64) and restricted life-space mobility (OR = 0.42, 95% CI: 0.26–0.67) were significantly associated with disability. Integrating frailty with life-space mobility evaluations demonstrated the highest predictive power for disability-related LBP (AUC = 0.89, 95% CI: 0.84–0.93). Conclusion: Frailty and restricted life-space mobility significantly predicted disability in older adults with LBP. Healthcare professionals should recognize the critical importance of integrating patient-reported outcomes with screening for frailty and life-space mobility limitation to optimize care or tract symptom progression.

## 1. Introduction

Low back pain (LBP) is a common health problem in musculoskeletal system diseases impacting people of all ages. The prevalence of LBP and disability related to LBP is reported to increase with age [1,2]. A high prevalence rate of LBP in older adults, ranging from 21.7% to 75.0% [3], which was reported to be 63.4% in Japan [4] and 42.4% in Brazil [5]. Similarly, in Thailand, the prevalence of LBP among older adults was 66.1% [6]. There is mounting evidence that advanced surgical treatment does not lead to full functional recovery in some cases, particularly in older adults; the majority of older adults with LBP remain dependent and require long-term care [1,3,4,5,6]. The ongoing aging of the population will continue to increase the likelihood of severe LBP-related consequences. Thus, early identification of potential risks and the promotion of proactive care that considers age-related decline are critical for providing better care and enhancing the quality of life in this population.

LBP can affect several aspects of life, including physical, psychological, and social impacts, in addition to the economic burden imposed by healthcare costs. The Global Burden of Disease study in 2017 reported that the number of years lived with disability was 64.9 million, and this value increased by 17.5% from 2007. Additionally, LBP is the most common cause of disability in both males and females [7]. Pain is the primary difficulty leading to physical limitation or disability for patients with LBP. It decreased mobility and ability to perform activities of daily living (ADLs) and can lead to stress, dependency, and long-term care, particularly in older adults [8,9]. Although several studies emphasized that LBP-related consequences and disability increase with age [1,2,3], a more concise understanding of the conditions of age-related decline in older adults with LBP is necessary to enhance care quality.

Because LBP has severe effects on the musculoskeletal system, older adults with age-related conditions may suffer more from physical limitation or disability caused by the rapid progression of musculoskeletal decline [1,2,3,8]. Recent evidence indicated that the age-related condition known as frailty—geriatric syndromes—significantly influences adverse health outcomes [10,11,12]. Frailty is a common geriatric syndrome clinically characterized by decreased reserve and resistance to stressors, both internal and external stressors, leading to accumulative decline across multiple physiologic systems and functions [10,11,12]. Hence, frailty may play an important role in decreasing the strength and endurance of musculoskeletal function and functional capacity [10], leading to decreased physical function. Moreover, frailty was found to be a risk factor for developing disability or permanent disability in the future [11,12]. Because of the complexity of frailty, both internal and external stressors may trigger frailty and exacerbate its severity [11,12,13,14]. Persistent pain was one of the stressors that caused frailty to occur [13], and pain is reported to be associated with increased risk and severity of frailty in older adults [14]. Recent studies have found that frail older adults with chronic pain exhibited greater limitations in ADLs and disability compared with older adults without frailty [15,16]. Therefore, frail older adults with LBP are more likely to develop functional limitations that lead to decreased strength and endurance, resulting in physical activity decline in both mobility and distance.

However, traditional measurements of mobility and physical activity do not provide measures for a comprehensive range of dimensions. The assessment of mobility is also limited by gaps in current knowledge, and there is a lack of measures for assessing distance-based mobility. The concept of life-space mobility has received increasing research interest and is used to assess individuals’ ability to perform movements across distance in daily life in various social contexts [17]. A number of studies have reported that reduced life-space mobility can predict adverse health outcomes such as morbidity, mortality, and hospitalization [18,19] and are associated with decreased physical performance, mobility limitations, and disability [20]. Older adults with chronic pain were reported to exhibit a higher prevalence of mobility limitations and to have more severe mobility limitations than those without pain [21]. In addition, during the coronavirus disease 2019 (COVID-19) pandemic, individuals in many countries were required to follow guidelines for preventing transmission of COVID-19, including social distancing and staying at home. These preventive measures may have increased the risk of mobility impairment or decline, leading to decreased life-space mobility during the pandemic. Older adults with LBP may have been more likely to be affected than other groups by limitations in healthcare access or limitations in daily life activity in this situation [22,23,24]. As previously indicated, older adults with LBP may experience negative impacts of functional decline and increased pain on mobility [11,12,13,14,15,16], resulting in decreased life-space mobility [20,21]. Therefore, assessment of the impact of LBP, including functional status, pain, and life-space mobility, is important for older adults with LBP.

Currently, the concept of patient-reported outcomes (PROs) has received increasing attention as an approach for assessing patients’ health status. PROs reflect the symptoms and severity of diseases, the impact of disease-related symptoms on ADL performance, physical and mental health status, health-related quality of life, and effective treatment experiences [25]. To improve ongoing care, continued monitoring of clinical outcomes is needed. In addition, because PROs are directly reported by patients, healthcare professionals can obtain quality information that is patient-centered and can be utilized for management and evaluation of treatment experiences, as well as identifying goals for treatment [25,26], eventually to enhance care quality and improve clinical outcomes. However, much less is known about how the conditions of age-related decline underpin disability in this population. In addition, the influence of frailty and life-space mobility on LBP-related disability remains unclear. The purposes of this study were to explore the prevalence of frailty, mobility restriction, and physical limitation and to investigate the predictability of frailty and life-space mobility on PROs of disability in older adults with LBP.

## 2. Materials and Methods

### 2.1. Study Design, Setting, and Participants

This research employed a cross-sectional analytical study design focusing on the main study variables of frailty, life-space mobility, and PROs of disability. With simple random setting selection, two tertiary hospitals located in Bangkok, Thailand, were selected.

The sample comprised older adults with LBP who visited at an orthopedic outpatient department at two tertiary care hospitals from December 2021 to February 2022. Eligible participants were recruited using purposive criteria: (1) aged 60 years and over, (2) diagnosed with LBP or degenerative disease of the lumbar spine but not scheduled for spinal surgery treatment and not within 3 months post-surgery, and (3) no lower limb disability or hemiparesis and not bedridden. Patients with cognitive impairment were excluded.

### 2.2. Sample Size

The sample size was calculated on the basis of Burmeister and Aitken’s [27] method using logistic regression analysis. The minimum sample size for calculation was determined to be at least 20 participants per variable. To enhance the predictability, we increased the sample to a minimum of 30 participants per variable on the basis of the magnitude of frailty. In this study, we examined five variables. Thus, the target sample size was 150 participants, and then the number was increased by 10% to account for dropouts and missing data. Therefore, the total target sample size in this study was 165 people.

### 2.3. Data Collection

The research was carried out by face-to-face interviews at the orthopedic outpatient departments or phone interviews if they were unavailable by the time during the hospital visits. The researcher screened the cognitive impairment of participants using the Six-Item Cognitive Impairment Test-Thai Version (6 CIT) [28]. In cases in which the participants did not present cognitive impairment (6 CIT scores < 8), the researcher started interviewing using standard measures and subsequently retrieved from medical records. The Demographic and Health Information Record Form was developed on the basis of a literature review. The form covered both personal (i.e., age, gender, and occupation) and health information (i.e., body mass index, history of smoking, and history of spine surgery) relating to LBP.

Modified Frailty Index 11 (mFI-11) is a scale for investigating frailty status [29]. The 11 items of the mFI-11 are each scored with a binary response (“yes” or “no”); “yes” responses correspond to 1 point, whereas “no” responses correspond to 0 points. In this study, the mFI-11 data were subsequently retrieved from medical records. The final mFI-11 scores range from 0 to 1 point. A score of greater than or equal to 0.27 points was used as a cut-off point that indicated frailty status, with higher scores indicating more severe frailty.

The Oswestry Low Back Pain Disability Questionnaire (ODQ)-Thai version was used to assess PROs of disability. The ODQ was translated into Thai by Sanjaroensuttikul [30]. This scale comprises 10 items related to daily activity. Each item has six possible statements (0–5 points); the total raw score is transformed into a percentage (multiplied by 2). Levels of physical limitation are classified into five categories: minimal disability (0–20%), moderate disability (21–40%), severe disability (41–60%), crippled (61–80%), and bed-bound or exaggerating symptoms/patient cannot perform physical activity by themselves (81–100%) [31].

The University of Alabama at Birmingham (UAB) Study of Aging Life-Space Assessment (LSA)-Thai version [32] was used to evaluate life-space mobility in older adults by referring to personal mobility during the preceding 4 weeks on the basis of the five distance levels of mobility with additional assessment of mobility patterns, frequency, the requirement of any assistance from another person, or an assistive device. The total score ranged from 0 to 120 points, with higher scores indicating the extent of life-space mobility with independent mobility and less requirement of another person or assistive device.

### 2.4. Statistical Analysis

Data were analyzed using RStudio (versions 2022.02.3). Descriptive statistics including frequency, percentage, mean, and standard deviation were calculated to describe patients’ demographic and health information, frailty status, life-space mobility, and PROs of disability. The PROs of disability (ODQ scores) were transformed to Z scores for standardization before robust logistic regression was performed to investigate the predictive power of frailty and life-space mobility on PROs of disability in older adults with LBP. The level of significance was defined as 0.05 (*p* < 0.05).

## 3. Results

### 3.1. Demographic Characteristics

All 165 older adults approached agreed to participate in the study. Among the participants, the majority were females, with an average age of 67.30 years (standard deviation [SD] = 4.58; range 60–84 years). Regarding general health information, body mass index values ranged from 14.69–39.54 kg/m^2^, with an average value of 25.88 kg/m^2^ (SD = 4.36). Of the participants, 90.91% had underlying diseases with at least one comorbidity; of these, nearly half had more than three diseases (44.85%). Regarding types of LBP, all participants experienced chronic LBP with an average score of 4.71 points (SD = 2.35). According to the frailty index, participants were classified as pre-frail (66.67%), non-frail (26.67%), and frail (6.66%). The ODQ Z-scores of PROs of disability for LBP indicated more than half of participants (56.36%) had minimal disability. The details of participants’ demographic characteristics and health information are shown in Table 1.

### 3.2. Frailty, Life-Space Mobility, and PROs of Disability

Table 2 shows the characteristics of frailty, life-space mobility, and PROs of disability. Regarding mobility, participants exhibited mobility restrictions, with composite life-space mobility scores ranging from 12 to 100 points, with average life-space mobility scores for level 1, 2, 3, 4, and 5 of 7.89 points (SD = 0.46), 15.41 points (SD = 2.12), 12.35 points (SD = 9.05), 15.36 points (SD = 9.24), and 5.32 points (SD = 6.09), respectively. Based on the ODQ assessment for PROs of disability, scores ranged from 2 to 62 points, with an average score of 27.21 points (SD = 14.05), indicating that participants exhibited poor physical function related to disability.

### 3.3. The Predictive Power of Frailty and Life-Space Mobility on PROs of Disability

In the robustness multiple logistic regression model, the impact of frailty and life-space mobility was examined, adjusting for age, gender, and pain score (Table 3). Frailty, life-space mobility, and pain score were significantly associated with the ODQ assessment for PROs of disability. For frailty, participants whose frailty score increased by one point had a 70% increased risk of developing severe disability (adjusted OR = 1.707, 95% confidence interval [CI]: 1.397–2.086, *p* < 0.001). Regarding life-space mobility, participants with no restricted independent life-space mobility who had a composite life-space mobility score one point higher than the average score (Z score = −0.01906 points) had a 58% lower risk of developing severe disability (adjusted OR = 0.422, 95% CI: 0.263–0.675, *p* = 0.003).

**Figure 1:**
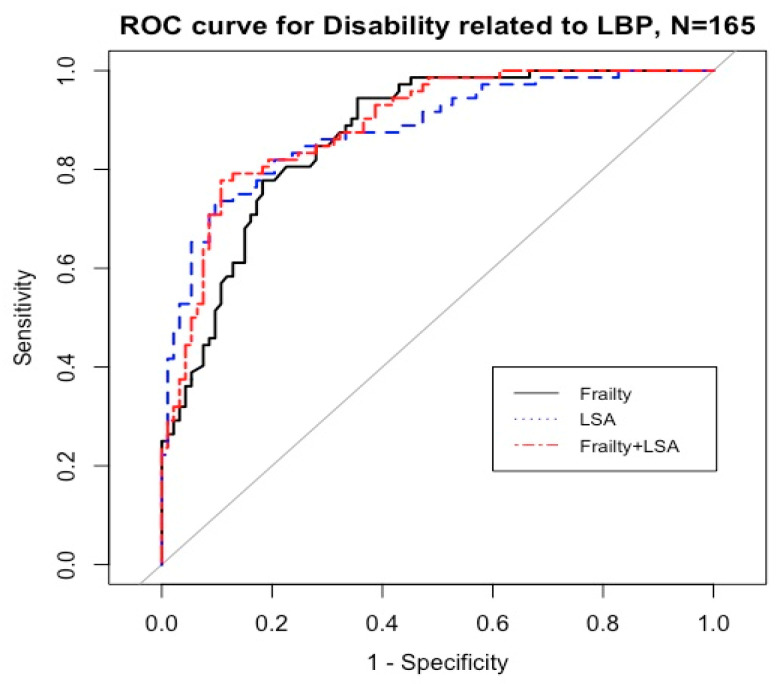
Predictive power of frailty and life-space mobility on the disability related to LBP. Abbreviations: ROC = receiver operating characteristics.

Comparisons of the area under the curve (AUC) were conducted for discriminating the predictive power of frailty and life-space mobility on disability related to LBP (Table 4, Figure 1).

## 4. Discussion

This study investigated the predictive power of frailty and life-space mobility on PROs of disability in older adults with LBP. In the present study, most participants were categorized as pre-frail and frailty. The prevalence of frailty in this study was lower than that reported by Kim et al. [33] in a study of 142 older adults with spinal stenosis. In that study, older adults with spinal stenosis were most commonly categorized as pre-frail (46.5%), followed by frail (41.5%) and non-frail (12.0%). Additionally, the results revealed that older individuals and those with spinal stenosis were 1.09-times and 14.35-times more likely to develop frailty compared with those who were younger and those without spinal stenosis, respectively. Compared with Kim et al.’s study, the participants in the current study had a lower average age (67.30 years) and had other diseases besides spinal stenosis. Additionally, our participants had a lower average ODQ score (27.21 vs. 36.90 points). A study by Tsuji et al. [16] investigated 730 community-dwelling older adults aged 65 years and older reported that individuals with increased ODQ scores were 1.05-times more likely to develop frailty. In addition, older participants were most commonly classified as pre-frail and frail (62.30%), with a minority classified as non-frail (37.70%). This finding may have occurred because all participants experienced chronic LBP (CLBP), which may lead to developing higher moderate to severe pain (63.64%). Similarly, the present study revealed that participants were more commonly classified as pre-frail and frail (73.33%) than non-frail (26.67%). These results were in accord with a previous study indicated that older adults with CLBP were more likely to exhibit pre-frail and frail status than older adults without CLBP [34]. In addition, chronic pain is a stressor that causes an imbalance in homeostasis for older adults and may stimulate frailty [13] as well as being associated with an increased risk and severity of frailty in both older men and women [14].

The mean life-space mobility score in the present study was lower than a previous study conducted on 156 older adults in orthopedic outpatient departments at two hospitals in Japan [35]. In that study, patients with frailty were 4.2-times more likely to exhibit restricted life-space mobility compared with those without frailty. Additionally, frailty can cause decreased muscle strength and muscle mass [10,36] leading to a decreased ability to perform ADLs and physical activity; consequently, life-space mobility may be increasingly restricted. In addition, most of the participants in the current study were females (73.94%), which accords with the findings of a previous study reporting that females exhibited significantly lower life-space mobility than males [35]. Nonetheless, the participants in this study had a mean life-space mobility score below 60 points, which is considered to indicate restricted life-space mobility [37]. This finding may have been partially caused by the COVID-19 pandemic, during which individuals were advised to follow preventive measures for preventing the transmission of COVID-19, including social distancing and staying at home. In Brazil, a study of 1482 community-dwelling older adults during the COVID-19 pandemic indicated that the mean life-space mobility score before the COVID-19 pandemic was 64 points, whereas that during the COVID-19 pandemic decreased to 37.8 points [38], indicating that older adults had restricted life-space mobility.

In the present study, the mean PROs of disability score were 27.21 points. According to the PROs of disability scores classified by level of disability, participants in the current study were most commonly classified as having moderate disability (46.07%). Likewise, a study was conducted on 126 patients with LBP who were admitted to the rheumatology ward at Bach Mai Hospital in Hanoi, Vietnam [39]. The results revealed that the average PROs of disability score were 26.39 points (SD = 10.48). Most patients had moderate disability (69.00%), which was a higher percentage of moderate disability than that observed in the present study. This may have occurred because most participants in that study had relatively high pain intensity, with 51.60% of participants exhibiting moderate pain and 42.10% exhibiting severe pain, and the participants were admitted to the hospital because of LBP symptoms; thus, they might have more severe symptoms than the participants in the current study, who were visiting the orthopedic outpatient department for follow-up treatment. In addition, the participants in the current study might have had appropriate pain management because there were no indications of severity requiring hospitalization, and our participants exhibited a lower proportion of pain intensity compared with that previous study—35.76% exhibiting moderate pain and 27.88% exhibiting severe pain. These findings are in accord with a previous report that pain was strongly positively associated with functional disability [9,40].

The current findings indicated that frailty and life-space mobility significantly predicted PROs of disability. Frailty exhibited the greatest predictive power on PROs of disability. Similarly, a study of 4349 older adults in China by Zhang et al. [41] revealed that older adults with pre-frail and frail status were 2.02-times and 4.10-times more likely to have an incidence of disability in ADL compared with those without frailty. These findings may have been caused by the greater prevalence of age-related decline among older people. Additionally, physiological reserves decline with age, and age is considered an important risk factor for developing frailty [10,42]. Furthermore, frailty increases weakness and reduces the ability to maintain homeostasis, resulting in the expression of clinical symptoms of frailty, such as unintentional weight loss, weakness, exhaustion, slow gait speed, and low levels of physical activity [43]. Additionally, pain has been reported to be correlated with physical limitations [9,21] and increased risk and severity of frailty [14]. Hence, frailty causes impaired functional ability leading to disability [11,12,13,14]. Moreover, the levels of pro-inflammatory cytokines increase with age and are related to the occurrence of frailty [44]. The pro-inflammatory cytokines also increase the catabolism of muscle, stimulating the loss of muscle mass and muscle strength and provoking the process of chronic disease and disability [44,45].

Restricted independent life-space mobility was significantly associated with PROs of disability in the current study. Our findings are in accord with those of a study of 755 community-dwelling older adults in Finland [46], which indicated that older adults with baseline life-space mobility scores of less than or equal to 52.3 points were 2.1-times more likely to develop difficulty in ADLs compared with those who had baseline life-space mobility scores exceeding 52.3 points (adjusted OR = 2.1, 95% CI: 1.2–3.7). These findings may have occurred because older adults with reduced life-space mobility exhibited more severe mobility limitations. Restricted life-space mobility causes decreased mobility and low physical activity, resulting in a risk of deterioration of physical performance in older people [20,47]. Older adults also exhibit age-related decline, such as the loss of muscle mass, muscle strength, and balance, which can cause immobility [36,43]. Additionally, chronic disease (e.g., diabetes mellitus) can cause increased loss of muscle mass and strength because of insulin resistance, which causes reduced protein synthesis and increased protein degradation. These factors can result in a decline in muscle mass [48].

Additionally, pain in older adults with LBP also affects mobility, and individuals with greater pain intensity are reported to be 1.26-times more likely to develop physical limitations [49]. Individuals with chronic pain had a 1.5-times higher risk of developing functional disability compared with those without chronic pain [50]. Furthermore, uncertainty regarding environmental factors, such as in the COVID-19 pandemic, may have involved further restrictions on life-space mobility among older adults [51] and may impact social network and support associated with uncertainty and quality of life among patients with chronic low back pain [52]. As mentioned above, reduced life-space mobility among older adults is associated with a decrease in muscle strength, independent mobility, and physical performance [53,54], resulting in an increased risk of developing physical limitations or disability in the future [46].

## 5. Conclusions

The findings revealed that older adults with LBP were more commonly classified as having pre-frail and frail status compared with non-frail status, and, overall, participants were identified as having restricted life-space mobility. Frailty and life-space mobility increased the risk of development of disability related to LBP and accurately predicted the patient-reported outcomes of disability. These findings suggest that healthcare professionals should integrate early detection of frailty and life-space mobility with the PROs of disability in enhancing care. Although the study has demonstrated encouraging findings, it has certain limitations. Due to the study design, some confounding factors, including pain characteristics, socioeconomic status, levels and types of spinal surgery, or emotional disturbance, may interfere with personal functional ability. Future studies on controlling these factors or exploring the nexus relationship underpinning frailty, life-space mobility, and disability related to LBP topics are therefore recommended. Moreover, to gain insight into illness trajectories in older adults with LBP, it may be more advantageous to develop further investigations of frailty and life-space mobility in various pain characteristics of LBP, such as acute, neuropathic, or chronic pain and its effect on patient-reported outcomes over the long term across different socio-cultural settings. In addition, the development of interventions to delay the progression of frailty along with promoting life-space mobility should be considered, to help maintain physical performance, enhance well-being, and lessen the likelihood of future disability in older adults with LBP.

## Figures and Tables

**Table 1:** Demographic characteristics and health information (N = 65).

Demographic Characteristics	Mean (SD)	N	%
Age (years)	67.30 (4.58)		
60–69		118	71.52
70–79		46	27.88
≥80		1	0.60
Gender			
Female		122	73.94
Male		43	26.06
Body Mass Index (kg/m^2^) *	25.88 (4.36)		
<18.5 kg/m^2^ (Underweight)		4	2.42
18.5–22.9 kg/m^2^ (Normal)		41	24.85
23.0–24.9 kg/m^2^ (Overweight)		29	17.58
25.0–29.9 kg/m^2^ (Obesity class I)		62	37.57
≥30.0 kg/m^2^ (Obesity class II)		29	17.58
Underlying diseases/Chronic diseases			
No		15	9.09
Yes **		150	90.91
Hypertension		110	73.33
Dyslipidemia		105	70.00
Diabetes mellitus		50	33.33
Cardiovascular diseases		18	12.00
Others		102	68.01
Number of comorbidities			
<3 diseases		91	55.15
≥3 diseases		74	44.85
History of spinal surgery			
Never		127	76.97
Yes		38	23.03
Types of low back pain			
Chronic low back pain		165	100.00
Pain intensity	4.71 (2.35)		
Mild pain (1–3 points)		60	36.36
Moderate pain (4–6 points)		59	35.76
Severe pain (7–10 points)		46	27.88
Frailty Classification			
Non-frail		44	26.67
Pre-frail		110	66.67
Frail		11	6.66
Patient-reported outcomes of disability			
Minimal disability		93	56.36
Severe disability		72	43.64

* BMI classifications for Asian population; ** Participant chose more than one disease.

**Table 2:** Mean scores of frailty, life-space mobility, and patient-reported outcomes of disability (N = 165).

Variables	Possible Range	Actual Range	Mean (SD)
Frailty			
Frailty (raw score)	0–10	0–5	1.19 (0.99)
Frailty (Frailty index converting score)	0–1	0–0.45	0.11 (0.09)
Life-space mobility			
Level 1	0–8	6–8	7.89 (0.46)
Level 2	0–16	0–16	15.41 (2.12)
Level 3	0–24	0–24	12.35 (9.05)
Level 4	0–32	0–32	15.36 (9.24)
Level 5	0–40	0–20	5.32 (6.09)
Life-space mobility (total scores)	0–120	12–100	56.32 (16.70)
Patient-reported outcomes of disability			
Minimal disability	0–20	2–20	13.07 (5.58)
Moderate disability	21–40	22–40	29.76 (5.53)
Severe disability	41–60	42–60	48.89 (4.97)
Cripple back pain	61–80	62–62	62.00 (0.00)
Bed-bound	81–100	-	-
Patient-reported outcomes of disability (total scores)	0–100	2–62	27.21 (14.05)

**Table 3:** Multiple logistic regression analysis among frailty, life-space mobility, and patient-reported outcomes of disability (N = 165).

Variables	B	Univariate ORs (95% CI)	*p* Value	B	Adjusted ORs (95% CI)	*p* Value
Age	0.069	1.071 (0.995–1.147)	0.052	0.080	1.083 (0.993–1.181)	0.070
Gender (Male)	0.228	1.255 (0.618–2.548)	0.529	−0.491	0.612 (0.237–1.581)	0.312
Pain score	0.494	1.639 (1.378–1.950)	<0.001	0.535	1.707 (1.397–2.086)	<0.001
Frailty	0.581	1.787 (1.261–2.533)	0.001	0.554	1.740 (1.146–2.641)	0.009
Life-space mobility scores	−0.834	0.434 (0.299–0.629)	<0.001	−0.863	0.422 (0.263–0.675)	0.003

Adjusted variables are age, gender, and pain score. Robustness multiple logistic regression was employed for analysis; CI = Confidence Interval; ORs = Odds ratios.

**Table 4:** The estimate of predictive power of frailty and life-space mobility on disability related-LBP among 165 older adults with LBP.

Predictors *	Disability Related-LBP (AUC)	95% CI
Frailty	0.868	0.816–0.921
LSA	0.880	0.828–0.933
Frailty + LSA	0.890	0.841–0.938

* Adjusting for age > 60, gender, and pain score; Robust logistic regression was analyzed; AUC = area under the curve; CI = confident interval; LSA = life-space mobility.

## Data Availability

Due to the nature of this research, participants of this study did not agree for their data to be shared publicly, so supporting data is not available.
